# Supplementary material for: Tryptophan Modulation in Cancer-Associated Cachexia Mouse Models
Source: Int J Mol Sci. 2023 Aug 21;24(16):13005. doi: 10.3390/ijms241613005 (PMC10455959; doi:10.3390/ijms241613005)
Supplement: Supplementary file 1 [file ijms-24-13005-s001.zip › ijms-2540719-supplementary.pdf]

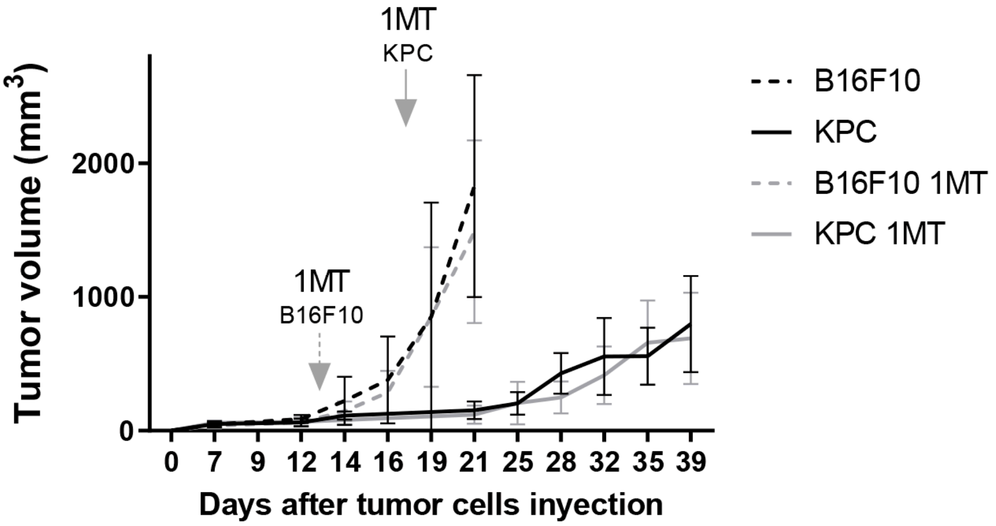

Figure S1. Tumor volume measured along the experiments. Tumor growth was monitored by digital calliper. Arrows indicate the time when 1-MT administration started.

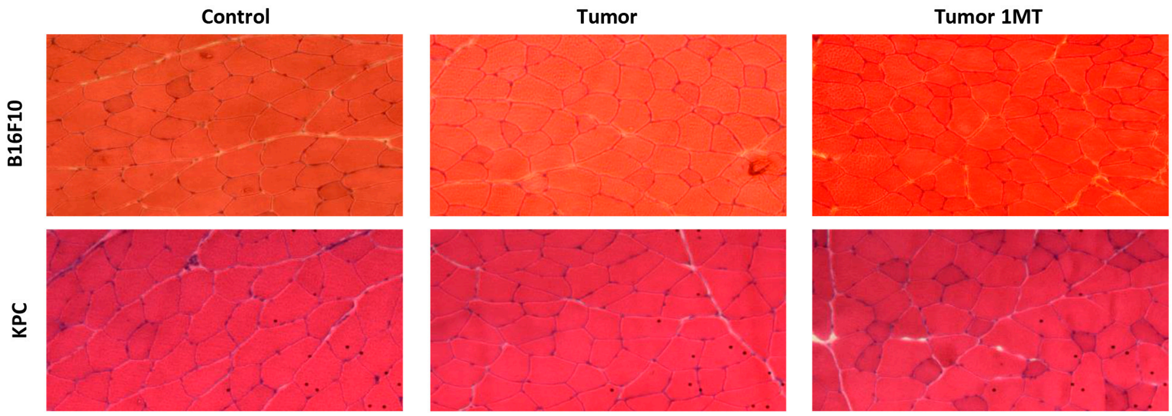

Figure S2. H&E staining representative of analyzed sections in our study. Frozen fresh quadriceps sections were stained using a standard H&E protocol. Representative pictures from the different conditions and models at the time of sacrifice is shown.

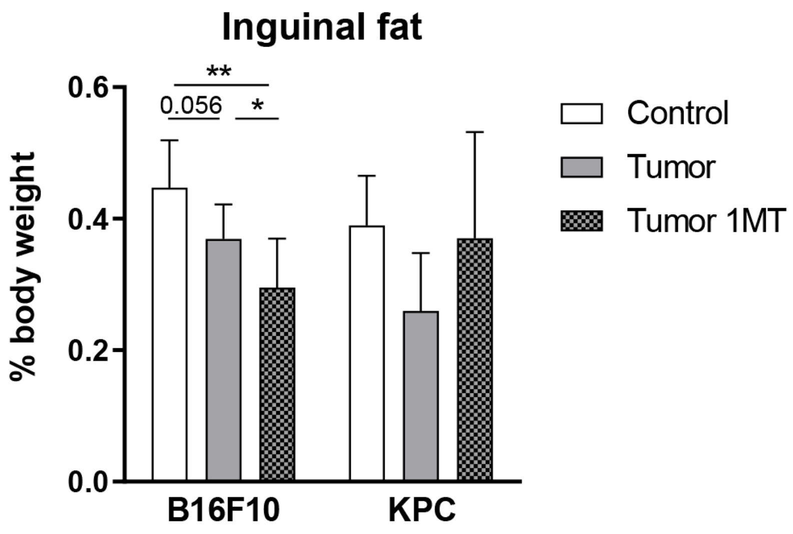

Figure S3. Inguinal fat weight at the time of sacrifice referred to total body weight. Significant differences between groups are indicated by * ($p < 0.05$) and ** ($p < 0.005$).
